# 

**Published:** 1842-12

**Authors:** 


### CONTENTS

OF NO. VI.

---

### ORIGINAL COMMUNICATIONS.

### ESSAYS AND CASES.

Art. I.—The Northern Lakes, a Summer Resort for Invalids of the South. By Daniel Drake, M. D., Professor in the Medical Institute of Louisville. - - 401

Art. II.—Remarks on the Diseases that appeared in Rutherford County, Tenuessee, during the year 1841,, and the winter of 1841-2; being a Report read before the Medical Society of Tennessee, at its annual meeting in May 1842. By John W. Richardson, M. D. - 426

Art. III.—A few practical Remarks upon Erysipelas of the Head. By Charles S. Tripler, M. D., Surgeon U. S. Army. - - - - - 439

### BIBLIOGRAPHICAL NOTICES.

Art. IV.—Elements of Surgery. By Robert Liston, Surgeon to the the North-London Hospital, Professor of Clinical Surgery, &c. &c. From the second London edition *with copious notes and additions,* by Samuel D. Gross, M. D., Professor of Surgery in the Louisville Medical Institute, &c. Illustrated with numerous engravings. Philadelphia: Ed. Barrington and Geo. D. Haswell; Louisville, James Maxwell, Jr. 1832. pp. 640, 8vo. - - - - - 453

Art. V.—The History, Diagnosis and Treatment of Typhoid and of Typhus Fever; with an Essay on the Diagnosis of Bilious, Remittent and of Yellow Fever. By Elisha Bartlett, M. D., Professor of the Theory and Practice of Medicine in Transylvania University. Philadelphia: Lea & Blanchard. 1842. pp. 393, 8vo. - - - - - - - 458
Art. VI.—Catalogue and Circular of the Medical Department of Laporte University. First Session, 1842. Laporte, T. A. Stewart. - - - - 460

SELECTIONS FROM AMERICAN AND FOREIGN JOURNALS.

Disease of the Kidney. - - - - - - 462
On the Blood-Globules, their Formation and Use. - 463
On the Coloration of the Bones by Madder. - - 465
Physiological Observations on Double Uterus. - - 467
Mode of Preserving Nitrate of Silver. - - - 467
External Application of Croton Oil. - - - 468
Nature and Treatment of Scrofula. - - - 468
State of the Blood in Hydrophobia. - - - 469
Asylum Journal. - - - - - 470
Case of Rupture of the Diaphragm in a Horse. - 470

ORIGINAL INTELLIGENCE.

Ourselves and our Enterprise. - - - - - 471
Transactions of the Alumni Society of the University of New York. - - - - - - 472
Sub-conjunctival Operation for Cataract. - - 473
Abuse of Calomel by Southern Physicians. - - 474

